# Horizontal-cell like Dm9 neurons in *Drosophila* modulate photoreceptor output to supply multiple functions in early visual processing

**DOI:** 10.3389/fnmol.2024.1347540

**Published:** 2024-05-15

**Authors:** Christopher Schnaitmann, Manuel Pagni, Patrik B. Meyer, Lisa Steinhoff, Vitus Oberhauser, Dierk F. Reiff

**Affiliations:** ^1^Department for Animal Physiology and Neurobiology, Institute of Biology I, Albert-Ludwigs-University Freiburg, Freiburg, Germany; ^2^Institute of Developmental Biology and Neurobiology, Johannes-Gutenberg-University Mainz, Mainz, Germany

**Keywords:** color vision, color opponency, photoreceptor, histamine receptor, horizontal cell, feedback inhibition, presynaptic calcium, insect

## Abstract

Dm9 neurons in *Drosophila* have been proposed as functional homologs of horizontal cells in the outer retina of vertebrates. Here we combine genetic dissection of neuronal circuit function, two-photon calcium imaging in Dm9 and inner photoreceptors, and immunohistochemical analysis to reveal novel insights into the functional role of Dm9 in early visual processing. Our experiments show that Dm9 receive input from all four types of inner photoreceptor R7p, R7y, R8p, and R8y. Histamine released from all types R7/R8 directly inhibits Dm9 via the histamine receptor Ort, and outweighs simultaneous histamine-independent excitation of Dm9 by UV-sensitive R7. Dm9 in turn provides inhibitory feedback to all R7/R8, which is sufficient for color-opponent processing in R7 but not R8. Color opponent processing in R8 requires additional synaptic inhibition by R7 of the same ommatidium via axo-axonal synapses and the second *Drosophila* histamine receptor HisCl1. Notably, optogenetic inhibition of Dm9 prohibits color opponent processing in all types of R7/R8 and decreases intracellular calcium in photoreceptor terminals. The latter likely results from reduced release of excitatory glutamate from Dm9 and shifts overall photoreceptor sensitivity toward higher light intensities. In summary, our results underscore a key role of Dm9 in color opponent processing in *Drosophila* and suggest a second role of Dm9 in regulating light adaptation in inner photoreceptors. These novel findings on Dm9 are indeed reminiscent of the versatile functions of horizontal cells in the vertebrate retina.

## Introduction

Color vision facilitates the identification of food sources, conspecifics, predators, and prey and supports a wide range of behaviors (Pfeiffer and Homberg, 2007; Osorio and Vorobyev, 2008; Cuthill et al., 2017; van der Kooi et al., 2020). It requires at least two photoreceptor classes with different spectral sensitivities and neural circuits that antagonistically compare their signals (Schnaitmann et al., 2020). Neurons that perform such comparisons are excited by certain types of photoreceptors and inhibited by others, resulting in color opponent responses that are a hallmark of color vision (Jacobs, 2008; Thoreson and Dacey, 2019).

Recent research demonstrates that color opponent processing emerges already in the presynaptic terminals of photoreceptors in the visual system of different vertebrates and insects (Schnaitmann et al., 2018; Thoreson and Dacey, 2019; Chen et al., 2020; Heath et al., 2020). Thus, evolutionary highly distant animals like monkeys and flies might employ similar circuit mechanisms to accomplish the first stage in color opponent processing.

In the retina of vertebrates, color opponent responses in photoreceptor terminals are mediated by spectral feedback inhibition from horizontal cells (HCs). HCs are inhibitory interneurons that laterally extend their processes into the outer plexiform layer to sample glutamatergic photoreceptor input. Light decreases glutamate release from vertebrate photoreceptors, reduces ionotropic glutamate receptor activation and thereby hyperpolarizes HCs. In turn, HCs then modulate glutamate release from their presynaptic input elements by parallel inhibitory feedback mechanisms, and are thought to provide feedforward inhibition to bipolar cells dendrites (Thoreson and Mangel, 2012; Diamond, 2017). By their feedback, HCs mediate surround inhibition of the characteristic center-surround receptive fields of photoreceptor terminals and ON- and OFF-bipolar cells, that enhance spatial and chromatic contrast (Werblin and Dowling, 1969; Baylor et al., 1971; Dacey et al., 2000; Thoreson and Burkhardt, 2003; Burkhardt et al., 2011; Crook et al., 2011). In addition, HCs are important players in “global” adaptation by reversible electric coupling. They form low-resistance electric networks and the extent of coupling is controlled by ambient light and circadian rhythm (Xin and Bloomfield, 1999; Chaya et al., 2017). Finally, HCs have also been implicated in locally restricted feedback inhibition to individual photoreceptor terminals (Chapot et al., 2017a). Because of their many functions in spectral processing and adaptation, HCs are considered multi-purpose interneurons of the outer vertebrate retina (Chapot et al., 2017b).

**Figure 1:**
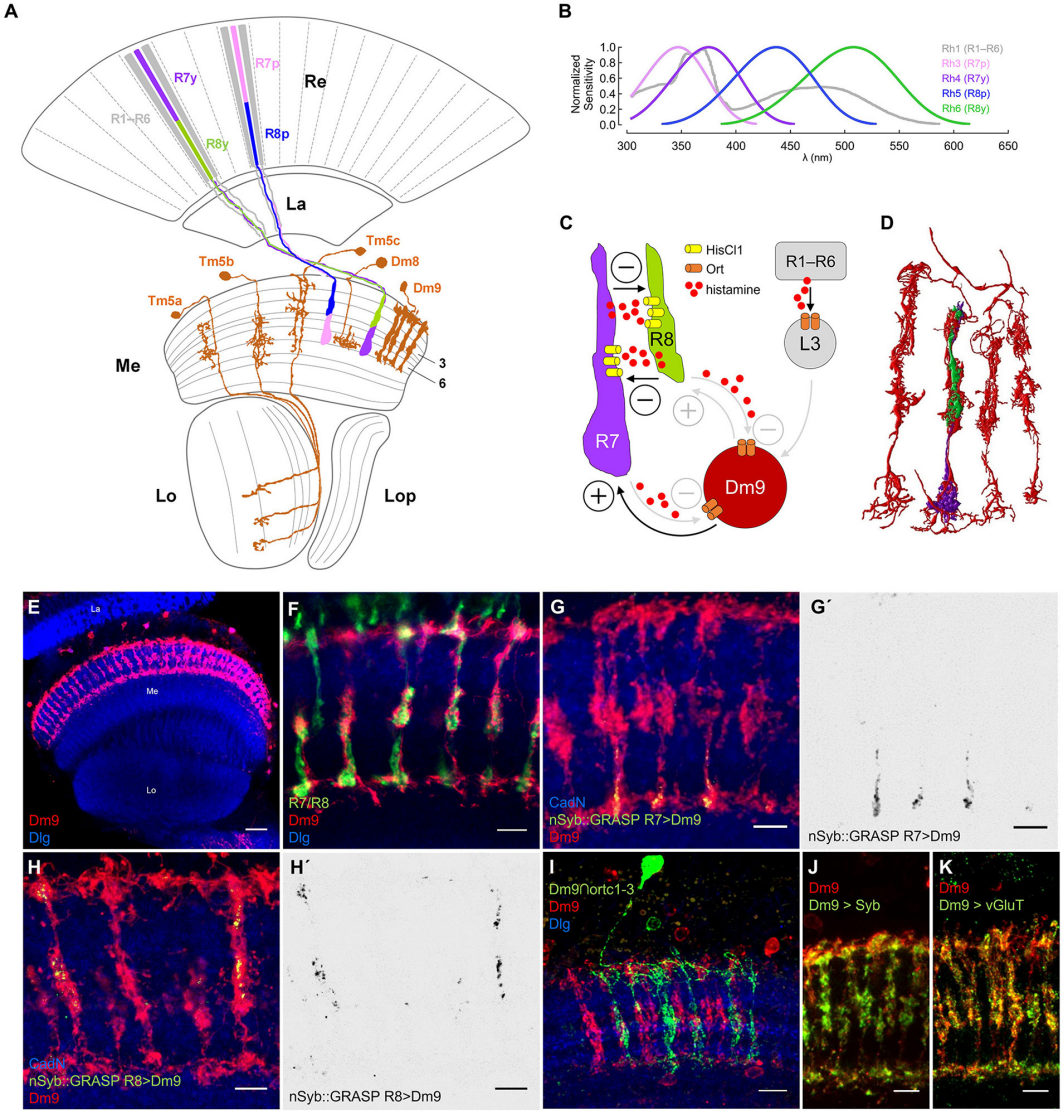
Dm9 neurons of the *Drosophila* visual system. **(A)** Schematic of the Drosophila optic lobe. The multicolumnar Dm9 cell and selected medulla projection and local interneurons postsynaptic to inner photoreceptors R7/R8 are shown in orange. R1–R6 outer photoreceptors (gray) project from the retina (Re) to the lamina (La). Yellow (y) and pale (p) inner photoreceptor tandems R7y/R8y (dark purple/green) and R7p/R8p (light purple/blue) project to the distal layers M1–M6 of the medulla (Me). R7p/R8p express rhodopsin rh3/rh5; R7y/R8y express rh4/rh6; R1–R6 express rh1 [Lo, lobula; Lop, lobula plate; adapted from Fischbach and Dittrich (1989)]. **(B)** Spectral sensitivity of the Rhodopsins expressed in the five major photoreceptor types (same color code as in **(A)**; data based on Salcedo et al., 1999). **(C)** Schematic of circuit interactions that shape the responses in R7/R8. Histamine release and HisCl1 receptors mediate mutual synaptic inhibition between R7 and R8 (Schnaitmann et al., 2018). Dm9 medulla neurons were proposed to mediate feedback inhibition to all R7/R8 photoreceptors (Heath et al., 2020). Based on serial EM analysis, R1–R6 transmit information to Dm9 via L3 lamina monopolar cells (Takemura et al., 2015; Davis et al., 2020; Kind et al., 2021). Demonstrated (black) and suggested (gray) excitatory and inhibitory synaptic connections indicated by (+) and (–), respectively. **(D)** Serial EM reconstruction of the neurites of Dm9 in four neighboring medulla column (red) and the terminals of a single R7/R8 pair (purple/green). Data from Takemura et al. (2015). **(E–K)** Confocal images of Dm9 cells in the optic lobe and the distal medulla layers M1–M6. Neuropil in blue (anti-Dlg or anti-CadN). Scale bars 5 μm, if not stated otherwise. **(E)** Dm9 neurons in the medulla expressing membrane tagged GFP (see methods, mCD8::GFP, anti-GFP, red), scale bar 30 μm. **(F)** Co-staining of Dm9 expressing Twitch-2C (anti-GFP, red) and R7/R8 terminals expressing DsRed (anti-RFP, green). **(G–H')** Activity-dependent, directional GRASP (nSyb::GRASP, anti-GRASP, green) highlights synaptic input from **(G, G')** R7yto Dm9 in M3-M6, and from **(H, H')** R8y to Dm9 in M1-M3. **(G', H')** Display the nSyb::GRASP signals alone. **(I)** Intersection of the ort-promoter-hemidriver (ort^C1 − 3^) and 64H01-GAL4 labels a single Dm9 cell expressing mCD8::GFP (anti-GFP, green) within the population of Dm9 expressing mCD8::RFP (anti-RFP, red). **(J)** Dm9 coexpressing the presynaptic vesicle marker nSyb::mRed (anti-RFP, green) and mCD8::GFP (anti-GFP, red).**(K)** Cell-specific expression of multi-epitope tagged vGluT (anti-FLAG, green) from a conditional allele highlights glutamatergic synaptic vesicles in Dm9 coexpressing mCD8::mCherry (anti-RFP, red).

In the fruit fly *Drosophila*, each of the ~780 ommatidia of the compound eye harbors six outer photoreceptors R1–R6 and a genetically determined tandem of pale (p) or yellow (y) inner photoreceptors R7/R8 (Figure 1A). Expression of rhodopsin 1 (rh1) and a UV-sensitizing photopigment make R1–R6 sensitive to a broad spectral range, from UV to green (Figure 1B; colors named according to human perception). R1–R6 transmit luminance information to the lamina, and enable downstream circuitries to detect visual motion and further achromatic visual features (Zhou et al., 2012; Borst and Groschner, 2023). However, R1–R6 additionally contribute to spectral processing and color vision (Schnaitmann et al., 2013; Li et al., 2021; Pagni et al., 2021).

R7/R8 project to the distal layers M1-M6 of the medulla where they convey the major input to the color vision circuitry. p and y R7/R8 are stochastically distributed over the main part of the eye (Figure 1A; Wernet et al., 2006). p R7/R8 express rh3/rh5 with maximum sensitivities in the short-UV/blue spectral range. y R7/R8 express rh4/rh6 with maximum sensitivity in the long-UV/green (Figure 1B; Salcedo et al., 1999; for photoreceptor sensitivity, see Schnaitmann et al., 2018; Heath et al., 2020; Sharkey, 2020). Two recent studies found that color opponent processing in R7/R8 terminals relies on a concerted action of two mechanisms. Mutual synaptic inhibition between R7 and R8 of the same ommatidium (same medulla column), and simultaneous feedback inhibition from postsynaptic neurons in the medulla, in particular multi-columnar Dm9 neurons that are post- and presynaptic to R7 and R8 (Schnaitmann et al., 2018; Heath et al., 2020) (Figure 1C). R7 and R8 terminals of an individual medulla column mutually inhibit each other via release of the neurotransmitter histamine and activation of postsynaptic HisCl1 receptors (R7/R8 axo-axonal inhibition) (Schnaitmann et al., 2018). To this point, p and y R7/R8 tandems can be considered spectral analyzers that preserve the spatial resolution of the eye. This is because the light-sensitive rhabdomeres of R7 and R8 are stacked on top of each other, both photoreceptors share the same optical axis, and they simultaneously sample the same spot in visual space. Spectral feedback inhibition that enables additional spectral comparisons that exceed pure p- and y-antagonism was suggested for all R7/R8 photoreceptor types, mediated by Dm9 cells (Heath et al., 2020). Dm9 ramify their neurites in the distal layers M1-M6 of ~7 neighboring columns of the medulla, where they closely intermingle with R7/R8 axons (Figures 1D–F). Electron microscopic reconstruction revealed that Dm9 are strongly connected to R7 and R8 being post- and presynaptic, and that Dm9 are postsynaptic to L3 lamina monopolar cells that relay luminance information from R1–R6 (Takemura et al., 2015; Ketkar et al., 2020; Kind et al., 2021). RNA-seq on Dm9 suggests that they use Ort-type histamine-receptors to detect histamine released by R7/R8 (Davis et al., 2020). Most important, calcium imaging experiments with genetic block of activity in Dm9 (cell-specific expression of Kir) or cell specific rescue of Ort receptor expression in Dm9 in histamine receptor mutant flies showed that Dm9 are required and sufficient for color opponent processing in R8p, respectively (Heath et al., 2020). Based on these findings on R8p and a computational model, it was proposed that Dm9 mediate similar spectral feedback inhibition in R8y, R7p, and R7y, and that Dm9 add an inhibitory surround to the ON-center responses of all R7/R8 (Heath et al., 2020).

Here, we employ a combined genetic and physiological approach to interfere with the functional properties of selected neurons in the early visual system of *Drosophila*, and to record calcium dynamics in same or different neuron types (Dm9 or terminals of inner photoreceptors R7/R8). Calcium recording is performed during visual stimulation of experimental animals using fly-back stimulation (see methods), targeted expression of the genetically encoded ratiometric calcium indicator Twitch-2C, and *in vivo* two-photon laser scanning microscopy (Reiff et al., 2010; Schnaitmann et al., 2018). We complement these experiments with the precise subcellular detection of key-proteins of synaptic function in genome-edited flies (Certel et al., 2022).

We report that activation of all four inner photoreceptor types elicits strong *ort*-dependent inhibition in Dm9, whereas activation of R1–R6 does not elicit detectable activity changes. This inhibition outweighs additional histamine-independent excitation of Dm9 by UV-sensitive R7 photoreceptors. Calcium imaging demonstrates that Dm9 indeed contribute to spectral feedback inhibition in all four inner photoreceptor types, as recently suggested. However, only in R7 terminals feedback from Dm9 is sufficient to generate color opponent responses. In terminals of R8, feedback inhibition from Dm9 must coincide with axo-axonal inhibition from the intra-ommatidial partner R7 to generate color opponent responses. Optogenetic inhibition of Dm9 that mimics Dm9′s responses to light, was associated with strong inhibition of all R7/R8 photoreceptor types. Cell-specific conditional labeling of multi epitope-tagged vGluT suggests that Dm9 release glutamate. Dm9 therefore likely mediates feedback inhibition by reducing excitatory glutamatergic input to R7/R8 in response to light increments. By this functional loop, Dm9 counteracts light induced depolarization in R7/R8 terminals. Finally, we report that persistent optogenetic inhibition of Dm9 abolishes color opponent processing in R7/R8 and shifts their sensitivity to higher light intensities. We propose that *Drosophila* Dm9 cells have important roles in both, color opponent processing and adaptation in presynaptic photoreceptor terminals, reminiscent of the versatile functions of HCs in the outer vertebrate retina.

## Materials and methods

### Flies

Flies were raised on standard cornmeal medium, 60% relative humidity, 14/10 h light/dark cycle, and 25°C. In our experiments we used female flies (white^+^) 2–6 days after eclosion. We used GMR21A12-GAL4 (Bloomington *Drosophila* stock center BDSC 48926), GMR56G04-GAL4 (BDSC 39161), GMR64H01-GAL4 (BDSC 39322), and GMR56G04-LexA (VK00027 on 3rd; Reiff Lab, this study) to drive expression in Dm9. UAS-Twitch-2C in attp40 (2nd) and LexAop-Twitch-2C in attp40 (2nd) or in VK00027 (3rd) were used in calcium imaging experiments (Schnaitmann et al., 2018; Pagni et al., 2021). For analysis of histamine signaling, we used *Hdc*^*JK*910^ (BDSC 64203), *ort*^1^*, ninaE*^1^ (BDSC 1133), *ort*^*us*2515^ (kindly provided by Pak Lab), *ort*^*P*306^*, hisCl1*^134^, and *ort*^1^*, ninaE*^1^*, rh6*^1^*, hisCl1*^134^ (kindly provided by Chi-Hon Lee) (Gao et al., 2008; Karuppudurai et al., 2014). We used hetero-allelic combinations because of additional mutations in *ninaE* and *rh6* (Karuppudurai et al., 2014; Schnaitmann et al., 2018). To restore ort expression, we employed UAS-ort (Rister et al., 2007). *rh1*-*norpA* (Salcedo et al., 1999), *rh3*-, *rh4*-, *rh5*-, and *rh6*-*norpA* (Wardill et al., 2012) were used to restore *norpA*-function in single photoreceptor types in *norpA*^7^ mutant flies (BDSC 5685). rh3-GAL4 (BDSC 7457), rh4-GAL4 (BDSC 8627), rh5-GAL4 BDSC 7458), rh6-GAL4 (BDSC 7459), rh3-LexA, rh4-LexA, rh5-LexA, and rh6-LexA (Schnaitmann et al., 2018; Pagni et al., 2021) were used to drive expression in R7p, R7y, R8p, and R8y. For optogenetic experiments, we expressed UAS-GTACR1 (Mauss et al., 2017); feeding of additional all-trans retinal was not necessary to inhibit Dm9. Mlp84B::GFP(3xP3-dsRed) was used to label photoreceptors (Vienna Drosophila Resource Center 318177). nSyb::GRASP was performed with LexAop-nSyb::spGFP1-10 and UAS-CD4::spGFP11 (BDSC 64315) (Macpherson et al., 2015). For promoter intersection we used ortc1-3-VP16^AD^ (Kindly provided by Chi-Hon Lee), 64H01-GAL4 (BDSC), and UAS-lexA^DBD^ (BDSC 56528) to drive LexAop-mCD8-GFP, UAS-mCD8-RFP (BDSC 32229). The mouse lymphocyte marker mCD8 is used to target GFP (or other chromophores) to the plasma membrane to improve surface labeling (Lee and Luo, 1999).

UAS-nSyb-mRed (Raghu et al., 2007) was used to label presynaptic sites in Dm9. For expression of a CRISPR/CAS9 generated conditional allele encoding multiple epitope tagged vGluT we used B2RT-STOP-B2RT-smFLAG-vGluT; 20xUAS-DSCP-B2/CyO_YFP;_ UAS-CD8::mCherry flies, kindly provided by Steven Stowers (Certel et al., 2022). For further information on genotypes refer to Supplementary Table S1.

### Two-photon calcium imaging

Flies were dissected and mounted for the recordings in Dm9 and R7/R8 terminals as described previously (Schnaitmann et al., 2018). In brief, flies were anesthetized by cold and bee wax was used to attach the thorax to a Plexiglas holder. Legs and head were fixed, the latter in a downward pointing position. Experimental flies were positioned underneath an aluminum holder that at the same time served as recording chamber. Two-photon imaging was performed through a small hole in the bottom of the recording chamber and head of the fly using a 40x, 1.0 NA water immersion objective (IR Plan-Apochromat; Zeiss), saline (103 mM NaCl, 3 mM KCl, 3 mM CaCl2, 4 mM MgCl2, 26 mM NaHCO3, 1 mM NaH2PO4, 10 mM trehalose, 10 mM glucose, 7 mM sucrose, 5 mM TES), and a custom two-photon laser scanning microscope (Schnaitmann et al., 2018). Fluorescence was excited using 823 nm light emitted by a mode-locked Ti:Sapphire laser (Tsunami, <100 fs, 80 MHz, 700–1,000 nm) pumped by a 10 W Millenia laser (both Spectra Physics), 5 to max. 20 mW at the specimen. Emission of mTurquoise2 and cpCitrine174 of Twitch-2C was recorded (50 × 50 pixels/image, 8 Hz) using BP 485/40, BP 535/30) and GaAsP photomultipliers (H10770PA-40 SEL, Hamamatsu) powered by a Sutter PS-2LV (Sutter Instruments, Novato, USA). Visual stimuli were presented during the return period of the x-scanning mirror (fly-back stimulation) (Reiff et al., 2010). Individual recording sequences were separated by minimum 40 s of darkness to warrant dark-adaptation of the eyes at the onset of recording.

### Visual stimulus presentation

Visual stimuli were presented as described in Schnaitmann et al. (2018). In brief, light from five spectrally different LEDs with maximum emission (in nm) at 517/green (APG2C1-515), 470/cyan (APG2C1-470), 430/blue (APG2C1-435), 375/UVlong (APG2C1-375-E) and 369/UVshort (APG2C1-365-E; Roithner Lasertechnik, Vienna, Austria) was focused on individual small light guides (105 μm core diameter, SFS105/125Y, Thorlabs, Newton, USA). These were combined into a single light guide (800 μm core diameter, FT800UMT, Thorlabs, Newton, USA) that was mounted in front of the fly's eye (Figure 1E). Emission of the green and cyan LEDs was shortpass filtered using FF01-533/SP (Semrock, Rochester, USA). Python 2.7 software and pulse-width modulation were used to control the timing and intensity of the LEDs over a range of four orders of magnitude (10^0^, 10^1^, 10^2^, and 10^3^ a.u.). The individual LEDs were calibrated for equal quantal flux at each intensity tested (high intensity (10^3^ a.u.) = 3.88 mmol s^−1^ m^−2^) and switched ON only during the fly-back of the x-scanning mirror (Reiff et al., 2010). This enabled visual stimulation at ~400 Hz, which is well-above the flicker-fusion frequency of the *Drosophila* eye and prohibited that photons of the wavelength-varying visual stimuli enter the detection pathway of the two-photon laser scanning microscope during functional recording. In GTACR1 experiments with delayed onset of laser scanning, the visual stimulus preceded the onset of scanning by 2 s. This enabled unperturbed R7/R8/Dm9 activity prior to the onset of scanning. In all “single stimulus” experiments, the order of the spectral stimuli was randomized and maximum intensity stimuli were presented, unless noted differently.

Color opponency in photoreceptor terminals was investigated by alternatingly presenting preferred monochromatic stimuli (R8y/green, R7y/UV_long_, R8p/blue, and R7p/UV_short_; intensity 10^1^ a.u., 2 s) and spectrally composite stimuli. In composite stimuli, the preferred wavelength of the recorded photoreceptor was combined with the individual other wavelengths, the latter typically with higher intensity (10^2^ a.u. for analysis of R8p/R8y, and 10^3^ a.u. for analysis of R7p/R7y; same as in Schnaitmann et al., 2018).

### Analysis of calcium imaging experiments

Potential lateral image motion was routinely compensated using image stabilization algorithms implemented in ImageJ. ROIs in single columnar elements of Dm9 and single photoreceptor terminals were defined by eye based on raw fluorescence. Changes of fluorescence were calculated after background subtraction (80%). The instantaneous fluorescence ratio over time R_t_ was calculated according to R_t_ = (intensity acceptor – 0.8 ^*^ background acceptor)_t_/(intensity donor – 0.8 ^*^ background donor)_t_. ΔR/R was calculated as (R_t_ – R_0_)/R_0_ with R_0_ being the average of 16 images (2 s) before stimulus onset. In GTACR1 experiments with delayed onset of two-photon imaging, R_0_ was calculated as the average signal in the final 4 s of the recording period. Bleach correction was performed on ΔR/R traces by subtracting an exponential decay functions fitted to the median of corresponding recordings without visual stimulation or by subtracting exponential decay functions fitted to the ΔR/R values before first stimulus onset and the last 4 s of each response trace (Schnaitmann et al., 2018; Pagni et al., 2021). ΔR/R response amplitudes were quantified by calculating the median during the last second of 2s visual stimuli and during the period from *t*_1_ = 2 s to *t*_2_ = 3 s of 5 s stimuli (GTACR1 experiments in Figure 3; Supplementary Figure S2). For stimulations with composite stimuli, inhibition or additional excitation of a wavelength stimulus added to a neuron's preferred stimulus was quantified by subtracting the response to the preferred stimulus from the response to the preceding composite stimulus (Schnaitmann et al., 2018). Imaging data were analyzed using MBF-ImageJ (US National Institutes of Health) and Python 2.7 software.

### Statistics

Statistical analysis was performed using SciPy (https://www.scipy.org/) and Python 2.7 software. All sample sizes were large enough for robust statistical tests. Groups that fulfilled the assumption of normal distribution (Shapiro–Wilk test) were tested for significant differences from zero using one-sample *t*-tests. Otherwise, we used non-parametric Wilcoxon signed-rank test. Prior to comparison of groups, we used both Shapiro-Wilk test for normal distribution and Levene test for equality of variances. Groups not significantly different from normal distribution and without significantly different variances were analyzed by one-way ANOVA followed by *post-hoc* pairwise two-sample *t*-tests. Otherwise, non-parametric Kruskall Wallis H test was performed, followed by *post-hoc* pairwise Mann-Whitney *U*-tests. *p*-values of all tests were corrected according to the Holm-Bonferroni method to control for the false discovery rate within multiple comparisons. Statistical details of experiments are described in the figure legends. We report number of recordings (*n*) and number of animals [in square brackets] in Supplementary Table S1. Since most data were not normally distributed, we plotted the median, 10/90%, and 25/75% quantiles to visualize data.

### Generation of transgenic flies

We generated GMR56G04-LexA using standard procedures. The 56G04 chromosomal region on the 3rd chromosome was amplified by standard PCR on cDNA from five heads of CantonS flies (Invitrogen) using the primers (left) “cagctctctgccactcagagaattt” and (right) “cggggtgcgtttgtaggctgatttc” that add 5′-AatII- and 3′-FseI-restriction sites. The resulting AatII/FseI fragment was cloned into pBPnLsLexA::GADflUw (kindly provided by Gerald Rubin, Addgene plasmid #26232). The construct was verified by sequencing. Transgenic flies were generated by phiC31mediated germline transfection in y1w^*^ P{nos-phiC31\int.NLS}X; PBac{y+-attP-9A}VK00027 (BDSC, 35569).

### Immunohistochemistry

Isolated brains were dissected in Schneider medium, fixed in 2% PFA for 50 min at room temperature, and washed four times in 0.5% PBT. The fixed brains were incubated first with PBT-NGS (3% Normal Goat Serum in PBT) for 60 min at room temperature and then with primary antibodies in PBT containing 0.05% sodium azide at 4°C overnight. Primary antibodies: mouse anti-GRASP (1:1,000, #G6539, Sigma), chicken anti-GFP (1:1,000, ab13970, Abcam), rat anti-RFP (1:200, #5f8, Chromotek), rat anti-CadN (3:100, DN-Ex #8, DSHB), mouse anti-dlg (1:30, 4F3, DSHB), and rabbit anti-FLAG (1:200, #14793, Cell Signaling Technology). After several washes with PBT, brains were incubated with secondary antibodies in PBT-NGS containing 0.05% sodium azide at 4°C overnight. Secondary antibodies: goat anti-mouse Alexa Fluor 488 (1:200, A-11001, Life Technologies), goat anti-rabbit Alexa Fluor 555 (1:200, A-21430, Life Technologies), goat anti-rat Alexa Fluor 405 (1:200, ab175671, Abcam), goat anti-mouse Alexa Fluor 633 (1:100, A-21053, Life Technologies), and goat anti-chicken Alexa Fluor 647 (1:200, ab150171, Abcam). After washing with PBT and PBS brains were mounted (Vectashield, Vector Laboratories) and optically sectioned with a Leica TCS SP8 STED 3X confocal microscope using HC PL APO 20x/0.75 IMM CORR CS2 and HC PL APO 63x/1.40 CS2 oil immersion objectives. Fiji software (https://fiji.sc/) was used for processing and analysis of confocal image stacks.

## Results

### Dm9 receive excitatory and inhibitory spectral inputs

In a first step, we set out to analyze input and output synapses in Dm9. We used LexA/LexAop to drive the expression of a non-fluorescent fragment of split GFP (spGFP^1 − 10^) fused to the presynaptic protein Synaptobrevin (LexAop-nSyb::spGFP1-10) in single types of photoreceptors. In same flies we used non-interacting Gal4/UAS to drive the expression of the missing fragment of GFP (spGFP^11^) fused to CD4 protein (UAS-CD4::spGFP11) in Dm9 cells. The CD4 domain anchors the non-fluorescent spGFP^11^ to the plasmamembrane of (here) Dm9. This approach is known as directional and activity-dependent GFP reconstitution across synaptic partners (nSyb::GRASP, Macpherson et al., 2015). If nSyb::spGFP1-10 expressing neurons establish chemical synapses on CD4::spGFP11 expressing neurons, both split-GFP fragments can locally reconstitute fluorescent GFP at chemical synapses. The reconstituted GFP can also be specifically detected by an highly selective antibody (Macpherson et al., 2015). Our experiments using nSyb::GRASP revealed strong deposits of reconstituted GFP indicating strong presynaptic input from all R7 and R8 to Dm9. The localization of nSyb::GRASP deposits indicates that R7- and R8-axons are presynaptic to Dm9 in the distal medulla layers M3–M6 with a focus on M6, and layers M1–M3, respectively (Figures 1G–H). These observations corroborate the results from serial-EM reconstruction (see also Figure 1D) and raise the question how light-induced activity changes in R7/R8 are detected in Dm9 (Takemura et al., 2015; Kind et al., 2021). Intersection of two hemi drivers, one containing the ort-promoter fragment *ort*^*c*1 − 3^ and the other active in Dm9, drove expression in Dm9 (Figure 1I). This suggests that Dm9 express *ort*, which is consistent with RNAseq data on Dm9 (Davis et al., 2020).

Because Dm9 are post- and presynaptic to R7/R8 (Takemura et al., 2015; Kind et al., 2021), we investigated the localization and identity of the neurotransmitter used in presynaptic terminals of Dm9. Expression of the presynaptic protein synaptobrevin fused to mRed (nSyb::mRed) labeled the neurites of Dm9 in all distal medulla layers M1-M6, with strongest labeling in M3 (Figure 1J). To explore the identity of the neurotransmitter that is likely released at these presynaptic sites, we employed a conditional multi-epitope tagged allele of the gene encoding the vesicular glutamate transporter vGluT (Certel et al., 2022). Cell-specific expression of this allele in Dm9 enabled the detection of endogenous vGluT throughout layer M1-M6 (Figure 1K). This result demonstrates the presence of a large number of glutamatergic synaptic vesicles in Dm9 and further supports the idea that Dm9 are glutamatergic (Davis et al., 2020). Glutamate release from Dm9 then likely excites R7/R8, as suggested by two recent studies (Davis et al., 2020; Heath et al., 2020).

Synaptic connectivity, expression of *ort* in Dm9, and calcium imaging suggest that Dm9 receives inhibitory input from all R7/R8 photoreceptor types (Heath et al., 2020). To test this assumption and whether inhibitory input fully explains the calcium responses exhibited by Dm9, we combined functional two-photon laser scanning microscopy with fly-back presentation of visual stimuli and expression of the ratiometric calcium sensor Twitch-2C in Dm9 (Reiff et al., 2010; Thestrup et al., 2014; Schnaitmann et al., 2018). In brief, in fly-back stimulation, the time used to present visual stimuli is limited to the brief return phase of the x-scanning mirror between the scanning of subsequent lines. It separates fluorescence recording and visual stimulus presentation in time and thereby prevents contamination of the recorded calcium imaging signals by stimulus light of changing wavelength.

**Figure 2:**
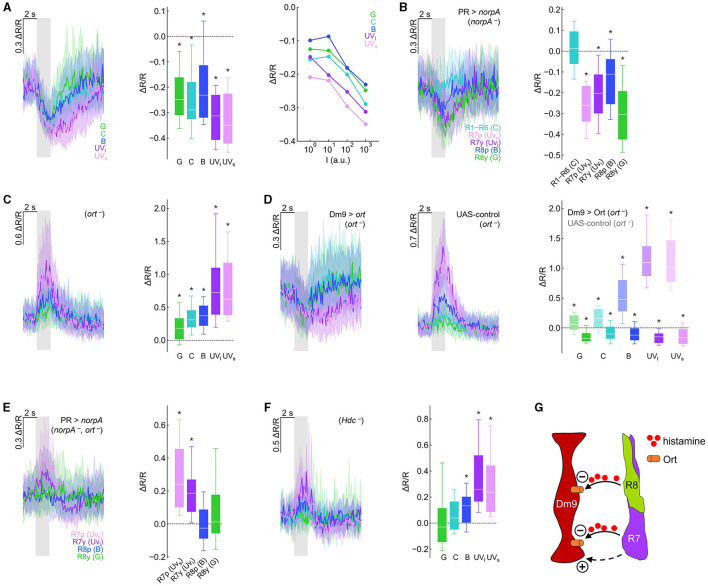
Genetic dissection reveals inhibitory and excitatory photoreceptor inputs to Dm9. **(A)** Calcium responses (ΔR/R) measured with the genetically encoded ratiometric calcium sensor Twitch-2C indicate a reduction in intracellular calcium in Dm9 neurons when visual stimuli are presented to the eyes of flies. (Left) Time course of responses to different isoluminant spectral stimuli, corresponding to maximum intensity stimuli. Responses to green, cyan, blue, UV_long_ (dark purple), and UV_short_ (light purple) are depicted with the respective color; gray box indicates stimulus period. (Middle) Quantification of the responses, *p < 0.05, Wilcoxon test. (Right) Median responses to color stimuli presented at four different intensities (10^0^-10^3^ a.u.). **(B)** Visually evoked responses in Dm9 in flies with only a single functional type of photoreceptor (*norpA* rescue), *p < 0.05, one-sample *t*-test. The wavelength of the presented visual stimulus always matched the maximum sensitivity of the rhodopsin expressed in the rescued photoreceptor type. **(C)** Dm9 responses in *ort*^−^ mutant flies, *p < 0.05, Wilcoxon test. **(D)** Dm9 responses in *ort*^−^ mutant flies with *ort* rescue in Dm9 (Dm9 > *ort, ort*^−^) and UAS-control flies that lack the Gal4-driver (UAS-ort, *ort*^−^, no GAL4), *p < 0.05, Wilcoxon test. Responses to the same light stimuli were significantly different between both genotypes, *p* < 0.05, Mann-Whitney *U*-test. **(E)** Dm9 responses in *ort*^−^ mutant flies with only a single functional photoreceptor type (*norpA* rescue, *ort* ^−^), *p < 0.05, Mann-Whitney *U*-test. **(F)** Dm9 responses in *Hdc*^−^ mutant flies, *p < 0.05, Wilcoxon test. **(G)** Schematic of the input signals to Dm9 (based on results in the Figure). (+) and (–) denote excitatory and inhibitory input, respectively. Dashed pathway indicates unknown histamine-independent excitatory input. Data are represented as median (solid line), 10%/90% quantiles (whiskers), and 25%/75% quantiles (box/error bands). Asterisks indicate responses significantly different from zero. If not stated otherwise, stimuli were shown at maximum intensity (10^3^ a.u.). For precise genotypes and number n recordings, see Supplementary Table S1. See also Supplementary Figure S1.

Light stimuli that together cover a wide range of wavelengths visible to the fly invariably inhibit Dm9 (Figure 2A; Supplementary Figure S1A). After light-OFF, the calcium responses returned to baseline without any signs of pronounced OFF-responses that were observed in a previous study (Heath et al., 2020). Furthermore, Dm9 calcium responses scaled with stimulus intensity over four orders of magnitude (Figure 2A).

To test whether each of the four inner R7/R8 and outer R1–R6 photoreceptors convey visual input to Dm9, we used *norpA*^−^ mutant flies. *norpA* encodes a phospholipase C, the key-enzyme of insect phototransduction and *norpA*^−^mutant flies have been widely used to interfere with the light sensitivity of fly photoreceptors (Inoue et al., 1985). We then rescued *norpA* expression in individual photoreceptor types (Wernet et al., 2012; Schnaitmann et al., 2013) and presented visual stimuli that matched the wavelength of maximum spectral sensitivity of the *norpA*-rescued photoreceptor type. Visual stimulation and functional rescue of either of the four types of inner photoreceptors reduced the calcium level in Dm9. In contrast, functional rescue and visual stimulation of R1–R6 did not result in detectable changes in the intracellular calcium level in Dm9. Thus, under our recording conditions, all four types of R7/R8 provide inhibitory synaptic input to Dm9 whereas R1–R6 do not (Figure 2B).

Building on these findings, we addressed whether light-induced activity in R7/R8 mediates inhibition in Dm9 that require *ort* receptors. If photoreceptor input to Dm9 is exclusively mediated by *ort* receptors, visual stimuli should not elicit any detectable responses in Dm9 in *ort*^−^ mutant flies. Contrary to this assumption, visual stimuli strongly increased intracellular calcium, suggesting strong excitatory responses in Dm9 of *ort*^−^ mutant flies, with strongest excitation to UV-light that is most efficiently detected by R7p and R7y (Figure 2C; Supplementary Figure S1B). Thus, Dm9′s inhibitory responses to light require Ort receptor expression and additional excitatory input to Dm9 is unmasked in the absence of Ort receptors.

To test whether expression of *ort* in Dm9 is sufficient to rescue the normally observed inhibitory responses, we targeted *ort*-expression specifically to Dm9 in otherwise *ort*^−^ mutant flies. This procedure restored the typical light-elicited inhibitory responses in Dm9 that were missing in UAS-control flies (that lack the Dm9-Gal4 driver) (Figures 2D; Supplementary Figure S1C). Thus, Ort receptors in Dm9 are sufficient to mediate inhibitory responses that outweigh additional Ort-independent excitation.

To reveal whether all four R7/R8 photoreceptor types provide excitatory input to Dm9, we recorded from Dm9 in *ort*^−^ mutant flies with only a single functional type of inner photoreceptor (photoreceptor-specific *norpA* rescue in *ort*^−^ mutant flies). Under these conditions, stimulus induced calcium responses in Dm9 were exclusively observed in flies with functional R7p and R7y, respectively (Figure 2E). Therefore, the *ort-*independent excitatory input to Dm9 originates from both R7 but not R8 photoreceptor types. We then tested whether excitatory input from R7 requires the release of histamine by recording Dm9′s calcium responses in *Hdc*^−^ mutant flies that lack functional histidine decarboxylase. In the absence of this enzyme, photoreceptors fail to synthesize the neurotransmitter histamine (Burg et al., 1993). However, in the absence of Hdc, Dm9 still exhibited increased calcium responses to visual stimuli (Figure 2F), indicating that excitation of Dm9 is mediated by histamine-independent signaling. This interpretation is further supported by experiments in which we recorded Dm9′s calcium responses in flies lacking both histamine receptors Ort and HisCl1. Also, in these double-mutant flies, Dm9 exhibited increased calcium responses to visual stimuli (Supplementary Figure S1D). In summary, all four types of inner photoreceptors convey robust histaminergic inhibition to Dm9 through the activation of Ort receptors expressed in Dm9. This inhibition counteracts and outweighs histamine-independent excitatory input from both R7 photoreceptor types via a so far unknown signaling mechanism (Figure 2G).

### Dm9 contributes to color opponency in all R7 and R8 photoreceptor types

**Figure 3:**
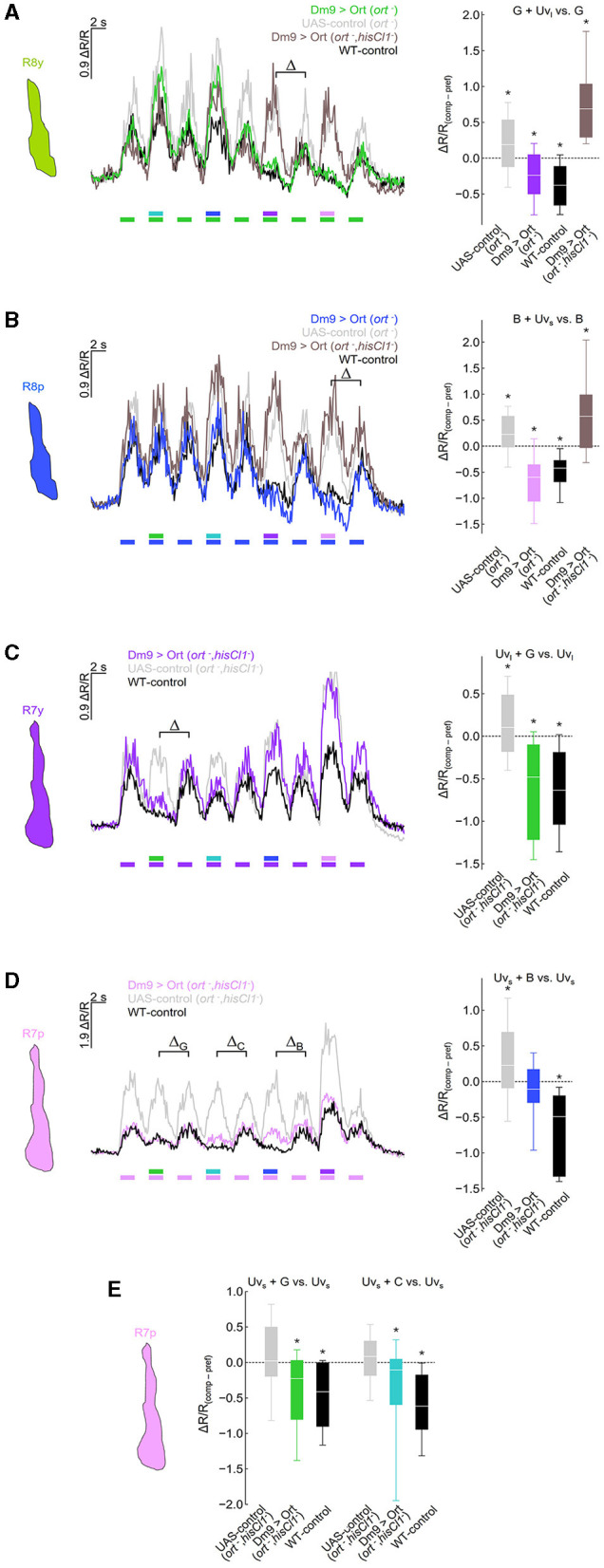
Dm9 mediates color opponent processing in all R7 and R8 photoreceptor types. **(A)** Responses of R8y photoreceptor terminals to opponent monochromatic stimuli in four different genotypes: flies with restored *ort* expression in Dm9 in *ort*^−^ mutant (colored line/box plot) or *ort*^−^*, hisCl1*^−^ double mutant flies (dark gray) harboring UAS-ort and Dm9-GAL4 driver, positive control flies (WT background; black), and negative UAS-control flies (*ort*^−^ mutants harboring UAS-ort but no GAL4; light gray). Stimulation consists of alternating presentation of photoreceptor type-specific preferred monochromatic stimuli and spectrally composite stimuli that additionally contain another monochromatic stimulus (stimulus protocols are shown below the recording traces; for intensities see methods). (Left) Time course of responses. (Right) Comparison of responses to preferred monochromatic and composite stimuli with UV_l_ (ΔR/R_comp_ – ΔR/R_pref_; indicated with the greek letter Δ in the left plot). Additive and subtractive (opponent) processing of the two wavelengths of composite stimuli is indicated by positive and negative values, respectively. ΔR/R_(comp−*pref*)_ of Dm9 > Ort (*ort*^−^) flies was significantly different from UAS-control (*ort*^−^) and Dm9 > Ort (*ort*^−^*, hisCl1*^−^) (statistical results and tests shown below). **(B)** Same as in **(A)** for R8p photoreceptor terminals. (Right) Comparison of responses to preferred monochromatic and composite stimuli with Uv_s_. ΔR/R_(comp−*pref*)_ of Dm9 > Ort (*ort*^−^) flies was significantly different from UAS-control (*ort*^−^) and Dm9 > Ort (*ort*^−^*, hisCl1*^−^) flies. **(C)** Responses of R7y photoreceptor terminals to opponent monochromatic stimuli in three different genotypes: flies with restored *ort* expression in Dm9 in *hisCl1*^−^*, ort*^−^ double mutants (colored line/box plot) harboring UAS-ort and Dm9-GAL4 driver, positive control flies (WT background; black), and negative UAS-control flies (*hisCl1*^−^*, ort*^−^ mutants harboring UAS-ort but no GAL4; light gray). (Right) Comparison of responses to preferred monochromatic and composite stimuli with green light. ΔR/R_(comp−*pref*)_ of Dm9 > Ort (*hisCl1*^−^*, ort*^−^) flies was significantly different from UAS-control (*hisCl1*^−^*, ort*^−^). **(D)** Same as in **(C)** for R7p photoreceptor terminals. (Right) Comparison of responses to preferred monochromatic and composite stimuli with blue light (indicated with the greek letter Δ_B_ in the left plot). ΔR/R_(comp−*pref*)_ of Dm9 > Ort (*hisCl1*^−^*, ort*^−^) flies was significantly different from UAS-control (*hisCl1*^−^*, ort*^−^) flies. **(E)** Comparison of responses to preferred monochromatic and composite stimuli with green or cyan light from data in [**(D)** left; indicated with the greek letter Δ_G_ and Δ_C_, respectively]. ΔR/R_(comp−*pref*)_ of Dm9 > Ort (*hisCl1*^−^*, ort*^−^) flies were significantly different from UAS-control (*hisCl1*^−^*, ort*^−^) flies. Asterisks indicate significant inhibition or additional excitation, *p < 0.05, Wilcoxon test. Significant difference between groups: *p* < 0.05, KruskalWallis *H*-test; *p* < 0.05, *post-hoc* Mann-Whitney *U*-tests. For genotypes and n recordings, see Supplementary Table S1.

Dm9 is proposed to mediate spectral inhibition in all R7 and R8 photoreceptor types, although solid physiological evidence exists only for R8p (Heath et al., 2020). To reveal whether Dm9 is indeed sufficient to mediate spectral inhibition in all R7 and R8 photoreceptor types, we conducted *ort* rescue experiments in Dm9 in *ort*^−^ histamine receptor mutant and *ort*^−^*, hisCl1*^−^ histamine receptor double mutant flies and recorded light-induced calcium responses in the terminals of each of the R7 and R8 types (Figure 3). In these experiments, color opponent processing in R8p/y was restored by *ort*-rescue in Dm9 in *ort*^−^ mutant flies, but not in the *ort*^−^*, hisCl1*^−^ double mutants. The restored color opponent responses match the responses in “wildtype” control flies, where R8p/y calcium responses to mixed wavelength stimuli (green + UV_long_ light in R8y, and blue + UV_short_ light in R8p) are smaller than the calcium responses elicited by the preferred wavelength alone, despite the strong increase in photon flux in the combined stimuli. In contrast, R8p/y calcium responses to mixed wavelength stimuli were larger than the calcium responses to the preferred stimulus alone, when *ort*-expression was rescued in Dm9 in *ort*^−^*, hisCl1*^−^ double mutant flies. The increased calcium responses indicate additive processing of the two stimulus components of mixed stimuli, similar to the results in UAS-control flies with *ort*^−^mutant genetic background (Figures 3A, B).

Similar experiments with recordings in R7y revealed that rescue of *ort* in Dm9 restored color opponent processing in the *ort*^−^*, hisCl1*^−^ double mutant. In contrast, R7y of UAS-control flies with double-mutant background processed green and UV_long_ additively (Figures 3C). Recordings in R7p in *ort*^−^*, hisCl1*^−^ double mutant flies revealed that *ort*-rescue in Dm9 partially rescues color opponent processing of blue and UV_short_. While color opponent processing of these stimuli was not fully rescued, *ort*-rescue clearly abolished the additive processing of mixed light stimuli that was again observed in the respective UAS-control flies (Figure 3D). However, R7p calcium responses in flies with ort-rescue in Dm9 exhibited significant spectral inhibition to green and cyan stimuli (Figure 3E). Together, these experiments demonstrate that Dm9 contributes to spectral inhibition in all R7 and R8 photoreceptor types. In R7p and R7y, Dm9 function is sufficient for color opponent processing, whereas R8p and R8y require additional spectral inhibition from the partner R7 via histamine and activation of HisCl1 receptors.

### Optogenetic inhibition of Dm9 prohibits color opponent processing in R7/R8

The experiments in Figures 2, 3 raise the question whether Dm9 function, i.e., light induced inhibition of Dm9 is required for color opponent processing in R7/R8. Color opponent processing in R7 is intact in both *ort*^−^ and *hisCl1*^−^ single-mutant flies respectively, suggesting that Dm9 function is not required for color opponency in R7 (Schnaitmann et al., 2018). Conversely, color opponent processing in R8 is abolished in *ort*^−^ and *hisCl1*^−^ single-mutant flies, respectively, suggesting that Dm9 is required for color opponency in R8 (Schnaitmann et al., 2018). Experiments with expression of Kir2.1 in Dm9 (Kir2.1 presumably hyperpolarized these cells) demonstrate that Dm9 is required for color opponent processing in R8p, while similar testing has not been performed in the remaining three inner photoreceptor types (Heath et al., 2020).

**Figure 4:**
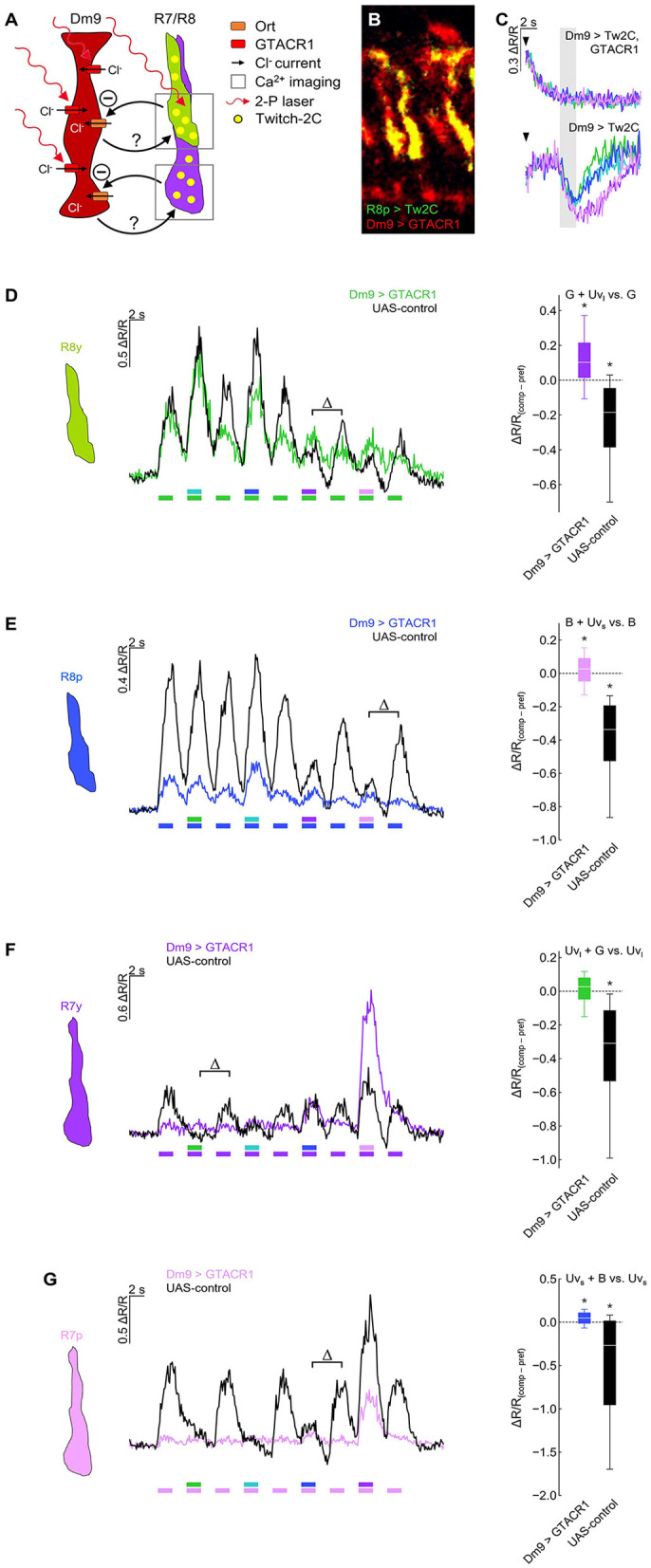
Optogenetic inhibition of Dm9 abolishes color opponent processing in R7/R8 photoreceptors. **(A)** Sketch of the experimental procedure to optogenetically inhibit Dm9 neurons during calcium imaging in inner photoreceptor terminals. Dm9 neurons expressed the light-gated chloride channel GTACR1 that we found to be activatable by the 823 nm two-photon laser [see **(C)**]. Note that R7/R8 input to Dm9 similarly causes chloride influx into Dm9 via Ort. Twitch-2C was expressed in single R7/R8 types. **(B)** Two-photon laser scanning image of R8p photoreceptors expressing Twitch-2C (green) and Dm9 neurons expressing EYFP-tagged GTACR1 (red) in the medulla (excited with 825 and 970 nm, respectively). **(C)** Laser scanning inhibits Dm9 neurons expressing GTACR1 and Twitch-2C. (Upper) Responses in Dm9 expressing GTACR1 after two-photon scanning onset (arrow head) and during presentation of color stimuli at maximum intensity (gray box). Note the strong activity decrease after scanning onset. (Lower) Responses in Dm9 lacking GTACR1 expression with same stimuli and scanning conditions. For quantification see Supplementary Figure S2A. **(D)** Responses of R8y photoreceptor terminals to opponent monochromatic stimuli in flies with GTACR1 expression in Dm9 (colored line/box plot) and UAS-control flies (harboring UAS-GTACR1 but no GAL4; black). Stimulation as in Figure 3. (Left) Time course of responses. (Right) Comparison of responses to preferred monochromatic and composite stimuli with UV_l_ (ΔR/R_comp_ – ΔR/R_pref_; indicated with Δ in the left plot). Additive and subtractive (opponent) processing of the two wavelengths of composite stimuli is indicated by positive and negative values, respectively. Asterisks indicate significant inhibition or additional excitation, *p < 0.05, Wilcoxon test. ΔR/R_(comp−*pref*)_ of Dm9 > GTACR1 flies was significantly different from UAS-control flies (*p* < 0.05, Mann-Whitney *U*-tests). **(E)** Same as in **(D)** for R8p photoreceptor terminals. (Right) Comparison of responses to preferred monochromatic and composite stimuli with UV_s_, *p < 0.05, Wilcoxon test. ΔR/R_(comp−*pref*)_ of Dm9 > GTACR1 flies was significantly different from UAS-control flies (*p* < 0.05, Mann-Whitney *U*-tests). **(F)** Same as in **(D)** for R7y photoreceptor terminals. (Right) Comparison of responses to preferred monochromatic and composite stimuli with green light, *p < 0.05, Wilcoxon test. ΔR/R_(comp−*pref*)_ of Dm9 > GTACR1 flies was significantly different from UAS-control flies (*p* < 0.05, Mann-Whitney *U*-tests). **(G)** Same as in **(D)** for R7p photoreceptor terminals. (Right) Comparison of responses to preferred monochromatic and composite stimuli with blue light, *p < 0.05, Wilcoxon test. ΔR/R_(comp−*pref*)_ of Dm9 > GTACR1 flies was significantly different from UAS-control flies (*p* < 0.05, *p* < 0.05, Mann-Whitney *U*-tests). For genotypes and n recordings, see Supplementary Table S1.

To address this question, we expressed the light-gated chloride channel GTACR1 (Govorunova et al., 2015; Mohammad et al., 2017) in Dm9 and recorded calcium responses in R7/R8 terminals (Figures 4A, B). Prior to these experiments, we recorded calcium responses in Dm9 that co-expressed GTACR1. In these Dm9 cells, we observed a strong decrease of the fluorescence ration ΔR/R, and thus a reduction in intracellular calcium that coincided with the onset of two-photon laser scanning. Thus, the 823 nm laser used for two-photon excitation of the donor chromophore of the genetic calcium indicator Twich-2C in addition efficiently activated GTACR1, without any need for additional optogenetic light-activation (Mardinly et al., 2018). Following the GTACR1 mediated inhibition, a further decrease or modulation of ΔR/R in Dm9 could not be detected when the eyes of flies were stimulated with light, even not at the highest intensity visual stimuli (Figure 4C; Supplementary Figure S2A). Thus, GTACR1-inhibition of Dm9 during two-photon calcium imaging in Dm9 reduced Dm9′s intracellular calcium to a very low level that likely prohibited a further decrease (or its detection) by light-activation of inner photoreceptors R7/R8 (Figure 2A). Alternatively, based on its function as light-activated chloride channel, GTACR1 might have increased intracellular chloride to a level at which the opening of Ort encoded histamine-gated chloride channels caused no relevant chloride flux.

We next tested whether color opponent processing in all inner photoreceptor types is altered when optogenetically inhibiting Dm9 (Figure 4). In these experiments, GTACR1-mediated inhibition in Dm9 prohibited color opponent processing in both R8 photoreceptor types, as expected (Figures 4D, E). Unexpectedly, GTACR1-inhibition of Dm9 similarly prohibited color opponent processing in both R7 photoreceptor types (Figures 4F, G). Color opponent processing in R7/R8 photoreceptor types was intact in all four UAS-controls (Figure 4). In addition, the calcium responses of all photoreceptor types of experimental flies with GTACR1 inhibition in Dm9 were decreased (Figure 4). Generally decreased calcium responses could in principle reduce or prohibit the detectability of spectral inhibition due to non-linearities of the calcium indicator and the impossibility of calcium levels smaller than zero nMol. However, this explanation can be dismissed as our observations not only indicate an absence of spectral inhibition but also revealed significant additive processing of composite stimuli in all photoreceptor types except for R7y. Together, these results demonstrate that intact function of Dm9 is required for color opponent processing in all types of inner photoreceptors.

### Inhibition of Dm9 shifts the dynamic range in R7 and R8

**Figure 5:**
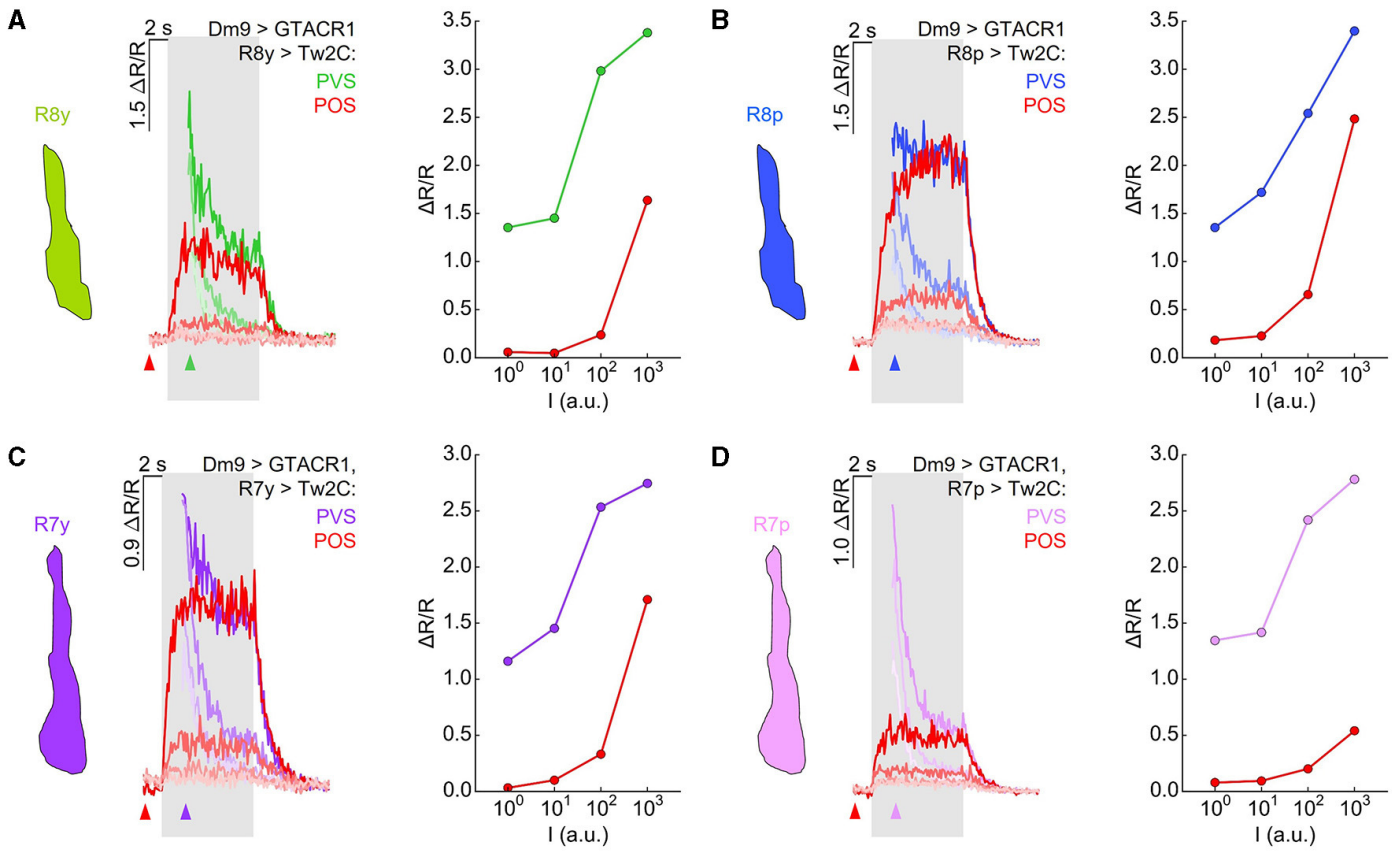
Optogenetic inhibition of Dm9 decreases calcium activity in R7/R8 photoreceptor terminals and shifts sensitivity toward higher stimulus intensities. **(A)** Responses in R8y to different intensities of green stimuli (10^0^-10^3^ a.u.) in flies expressing GTACR1 in Dm9. In one set of experiments, the onset of calcium imaging and optogenetic stimulation (elicited by the two-photon laser used for calcium imaging) precedes visual stimulus presentation (red; “preceding optogenetic stimulation”, POS; onset of laser scanning indicated by red arrow head). In the second set of experiments, the visual stimulation precedes the onset of calcium imaging and optogenetic stimulation [green; “preceding visual stimulation”, PVS; onset of laser scanning indicated by colored (green) arrow head]. Gray box indicates visual stimulation period. The magnitude of the responses correlates with stimulus intensity. (Left) Time course of responses. (Right) R8y average responses to the color stimuli presented at four different intensities (10^0^-10^3^ a.u.) calculated between 2 s and 3 s after visual stimulus onset (responses with and approx. without Dm9 inhibition). **(B)** Same as in **(A)** for R8p with blue stimuli. **(C)** Same as in **(A)** for R7y with UV_l_ stimuli. **(D)** Same as in **(A)** for Ryp with UV_s_ stimuli. Responses to the same intensity stimuli differed between the two experimental groups in **(A–D)**, *p* < 0.05, KruskalWallis *H-*test; *p* < 0.05, *post-hoc* Mann-Whitney *U*-tests. For genotypes and n recordings, see Supplementary Table S1. See also Supplementary Figure S2.

Activation of Dm9 by the light-gated cation channel CsChrimson caused excitation in R7 terminals, indicating an excitatory, sign-conserving synapse from Dm9 onto R7 (Heath et al., 2020). Together with the inhibitory, sign-inverting synapse (arthropod photoreceptors are excited by light ON) from R7/R8 onto Dm9, this processing is consistent with a role of Dm9 in providing inhibitory feedback to R7/R8. To further analyze the role of Dm9 in feedback inhibition, we optogenetically inhibited Dm9, which mimics the calcium response of Dm9 to light ON, and recorded the calcium responses in R7/R8 photoreceptors to visual stimuli with intensities ranging over four orders of magnitude. The presented stimuli had a duration of 5 s and their wavelength matched the maximum sensitivity of the recorded photoreceptor type. In one type of experiments, optogenetic inhibition of Dm9 starts with the onset of two-photon laser scanning that preceded the onset of the visual stimulus by 2 s (Figure 5). Under these conditions, presentation of light stimuli at the two lowest intensities elicited only minimal calcium responses in all R7/R8 photoreceptor types. Calcium responses to stimuli at the second highest intensity level were still unexpectedly small, while stimuli at the highest intensity elicited larger calcium responses (Figures 5A–D, “preceding optogenetic stimulation”, reddish traces). Thus, inhibition of Dm9 can strongly decrease or even eliminate the terminal calcium responses in R7/R8 to light.

To reveal stimulus induced calcium responses in R7/R8 that approximate the calcium responses in the absence of GTACR1 activation in same flies, we presented visual stimuli 2 s in advance of the onset of two-photon calcium imaging and thus 2 s before the onset of optogenetic inhibition of Dm9 (Figures 5A–D, “preceding visual stimulation”, cell type-specific colored traces). In these experiments, the calcium responses recorded at the onset of functional imaging roughly approximated the magnitude of the calcium responses that were observed in the absence of GTACR1 expression. However, immediately following the onset of two-photon imaging, R7/R8 calcium responses dropped rapidly. The time course of these calcium responses is consistent with a vivid onset of optogenetic inhibition of Dm9 by GTACR1 and feedback inhibition from Dm9 to R7/R8 (Figure 4C). The calcium responses then rapidly reached the level that we previously observed in experiments with “preceding optogenetic stimulation” (Figures 4D–G). To rule out that the observed effect is due to a potential leaky expression of GTACR1 in other neural cell types, we conducted the same experiments in UAS-control flies that lacked the Dm9-GAL4 driver. In these flies, inner photoreceptor calcium responses with “preceding optogenetic stimulation” were similar to their calcium responses with “preceding visual stimulation” (Supplementary Figure S2). Altogether, these experiments demonstrate that inhibition of Dm9 can dramatically reduce the calcium responses in R7 and R8 terminals. Notably, R7 and R8 photoreceptors appear to be not simply suppressed. Rather, their dynamic range is shifted toward higher light intensities, which is in accordance with an additional role of Dm9 in light-adaptation of inner photoreceptors.

## Discussion

Inner photoreceptors R7/R8 transmit the major signals to the color vision circuitry in *Drosophila* and recent calcium imaging studies demonstrated that R7/R8 spectrally inhibit each other by two parallel circuit mechanisms (Schnaitmann et al., 2018, 2020; Heath et al., 2020). First, reciprocal inhibition occurs between R7 and R8 photoreceptors of the same ommatidium, mediated by the neurotransmitter histamine that triggers the activation of HisCl1 receptors. Second, neurons of the medulla detect R7/R8 input and provide feedback inhibition to R7/R8 (Schnaitmann et al., 2018; Heath et al., 2020). Multicolumnar Dm9 cells that sample the input from several R7 and R8 were identified as the medulla neurons that convey feedback inhibition to R8p (Heath et al., 2020). Based on combinatorial *norpA*-rescue experiments, serial EM-analysis, and RNA-seq, this function of Dm9 was conferred to all four types of inner photoreceptors and pivotal for a computational model used to explain color opponent processing in the peripheral visual system of the fly (Takemura et al., 2015; Davis et al., 2020; Heath et al., 2020; Kind et al., 2021).

Here, we combined *in vivo* two-photon calcium imaging with genetic circuit manipulation to further elucidate the role of Dm9 in early visual processing. Our results demonstrate that all four types of inner photoreceptors R7/R8 convey visual input to Dm9. Dm9 cells exhibit reduced calcium responses indicative of inhibition to all tested spectral stimuli that scale with stimulus intensity (Figures 2A, B). However, the calcium responses of Dm9 were more complex than expected. R7 and R8 provide strong inhibitory histaminergic input to Dm9 via Ort receptor activation. This histamine-/Ort-mediated inhibition outweighs additional, previously unrecognized excitatory input from R7 that does not involve the neurotransmitter histamine. The physiological role of excitatory input from UV-sensitive R7 remains to be analyzed (Figures 2C–F).

**Figure 6:**
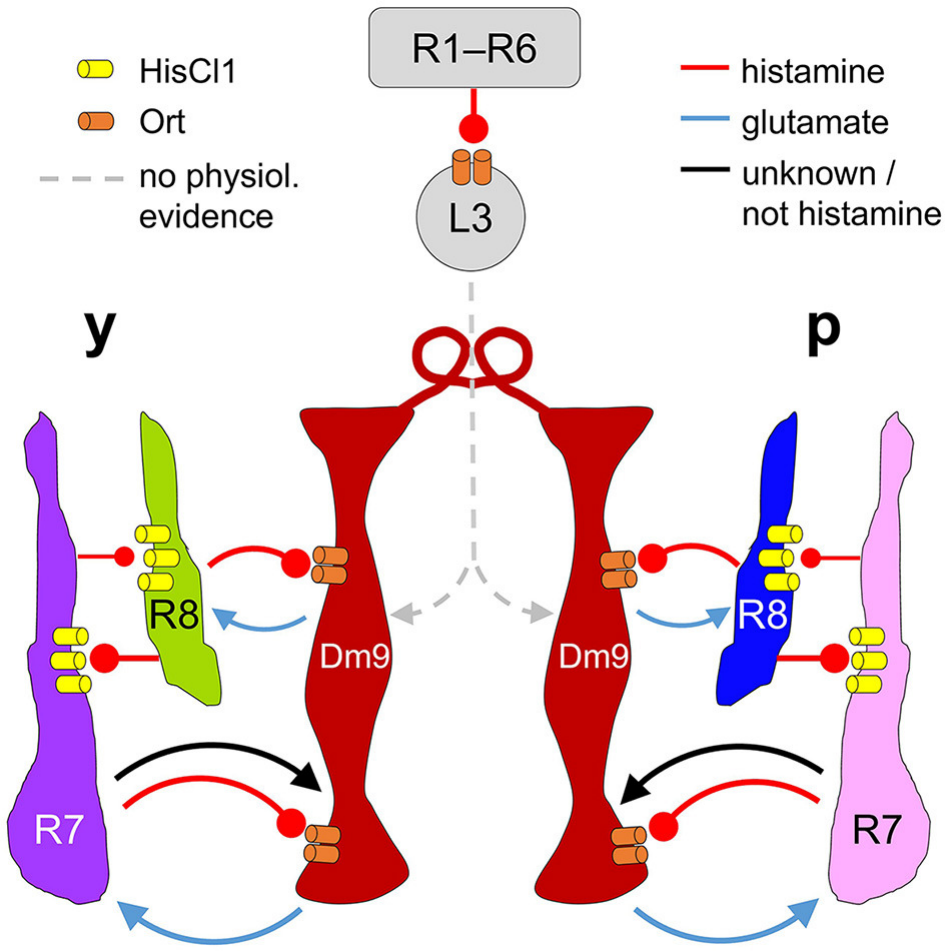
Proposed circuit diagram for the multiple interactions between R7, R8, and Dm9. Light depolarizes R7/R8 photoreceptors and increases the release of histamine. R7/R8 of the same ommatidium and medulla column mutually inhibit each other via histamine and HisCl1 receptors. In parallel, all four types of inner photoreceptors R7/R8 convey inhibitory input to Dm9 cells (only two columns shown) via histamine and its receptor Ort. R7 photoreceptors also provide excitatory input to Dm9, which is outweighed by inhibition. Dm9 cells then reduce the release of glutamate thereby decreasing excitatory input to the photoreceptors. Because single Dm9 cells receive p and y input in several neighboring columns, this processing establishes feedback inhibition that goes beyond purely p and y interactions, and is supposed to provide an inhibitory surround to individual presynaptic photoreceptors. Additionally, Dm9's feedback inhibition provides an important contribution to the regulation of the overall light sensitivity in inner photoreceptors R7 and R8. Physiological evidence for synaptic input to Dm9 from R1–R6 photoreceptor signal-transmitting L3 lamina neurons, revealed by EM, has not yet been shown. Arrows and oval arrows indicate excitatory and inhibitory signaling.

Our results demonstrate that Dm9 cells indeed have a key role in color opponent processing in all four R7/R8 photoreceptor types, as previously suggested (Heath et al., 2020). This conclusion is substantiated by different calcium imaging experiments. First, Ort-rescue in Dm9 demonstrates that histaminergic signaling from R7/R8 is sufficient to mediate spectral inhibition in both R7 types, whereas the same processing in both R8 types requires Dm9 in addition to simultaneous activation of HisCl1 receptors in R8 by direct inhibition from the partner R7 (Figure 3). Second, optogenetic inhibition of Dm9 abolishes color opponent processing in all four types of R7/R8 (Figure 4). Notably, we report that optogenetic inhibition of Dm9 strongly decreases the calcium responses of inner photoreceptors R7/R8 to light and shifts their sensitivity to higher light intensities (Figure 5). Because optogenetic inhibition mimics the effect of light, we propose that Dm9 simultaneously contribute to the adaptation of inner R7/R8 to ambient light. Such a dual role would make Dm9 even more “horizontal cell-like” than previously thought (Figure 6) (Davis et al., 2020; Heath et al., 2020; Uhlhorn and Wernet, 2020).

### Histamine-dependent and -independent photoreceptor signaling to Dm9

The observed histaminergic input from all R7/R8 photoreceptor types to Dm9 is in line with the reported strong synaptic connections between these cells that were revealed by serial EM-analysis (Takemura et al., 2015; Kind et al., 2021). According to these studies, Dm9 are additionally postsynaptic to lamina L3 neurons that receive strong synaptic input from the outer photoreceptors R1–R6 (Takemura et al., 2015; Davis et al., 2020). However, under our experimental conditions, light activation of R1–R6 did not elicit any detectable calcium responses in Dm9 (Figure 2B), which is consistent with results of previous studies that did not find any contribution of R1–R6 to color opponent processing in R7/R8 (Schnaitmann et al., 2018; Heath et al., 2020). Whether and how R1–R6 and L3 contribute to processing in Dm9 therefore remains unknown.

Activation of R7 by light elicited excitation in Dm9 in the absence of both histamine synthesis and histamine receptor expression (Figure 2). R8 cells have recently been implicated in (histamine-independent) excitatory cholinergic signaling to medulla neurons, in particular of the circadian clock circuitry in the medulla (Alejevski et al., 2019; Pagni et al., 2021; Xiao et al., 2023). Do R7 photoreceptors also use cholinergic signaling? Reports on the expression of choline acetyltransferase (ChAT) in R7 are controversial (Davis et al., 2020; Xiao et al., 2023). The study of Davis et al. (2020) reported low amounts of mRNA encoding the vesicular acetylcholine transporter (VAChT) in R7 and mRNA encoding ACh-receptors in Dm9 (Davis et al., 2020; Xiao et al., 2023). Similarly, sign conserving electrical synapses or ephaptic signaling could be at work, as reported for horizontal cell feedback to vertebrate photoreceptors (Thoreson and Mangel, 2012; Chapot et al., 2017b; Diamond, 2017). Notably, ephaptic signaling has also been described between R1–R6 and lamina monopolar cells of the fly visual system (Weckström and Laughlin, 2010).

### Parallel circuit mechanisms mediate color opponency in R7/R8

Previous studies as well as our new results demonstrate that color opponent processing in all inner photoreceptor types is mediated by two parallel circuit mechanisms (Schnaitmann et al., 2018; Heath et al., 2020). These mechanisms are not redundant. The HisCl1-dependent reciprocal inhibition between R7/R8 generates p- and y-specific opponent processing that retains the resolution of the compound eye (Schnaitmann et al., 2018). In contrast, Dm9 and Ort-dependent feedback inhibition enables additional inter-ommatidial processing in R8p and possibly all inner photoreceptor types (Heath et al., 2020). Based on its multicolumnar ramifications (Figure 1), Dm9 was furthermore proposed to mediate spatial surround-inhibition in inner photoreceptors (Heath et al., 2020). However, initial experimental analyses of the spatial layout of the receptive field of inner photoreceptors revealed simple ON receptive fields and no evidence for classical ON-center/OFF-surround receptive fields (Li et al., 2021).

Interestingly, the two mechanisms operate with different strength in R7 and R8. *ort* expression in visual circuits and *hisCl1* expression in R7, respectively, are each sufficient to mediate color opponent processing in R7 terminals. In contrast, color opponent processing in R8 requires simultaneous *ort*-mediated signaling to medulla circuits together with *hisCl1*-mediated inhibition from the partner R7 (Schnaitmann et al., 2018). In line with these results and the experiments on R8p performed by Heath et al. (2020), we here report that Dm9 is required for color opponent processing in both R8 photoreceptor types (Figure 4). In contrast to our results, Heath et al. (2020) report that *ort* expression in Dm9 is sufficient for color opponency in R8p and that additional HisCl1 is not required. We here investigated sufficiency and requirement of Dm9 for color opponent processing in R8p and in the remaining R7/R8 photoreceptor types. In agreement with our previous results, rescue of *ort* expression in Dm9 in *hisCl*1^−^ mutant flies is sufficient for color opponency in R7 but not in R8 that additionally require HisCl1 (Figure 3). Such functional differences might be caused by differences in the presented light stimuli, in particular intensity, spectral composition, background illumination and the adaptational state of experimental flies.

We furthermore observed an apparent inconsistency in our results on the requirement of Dm9 in color opponent processing in R7. Our previous work on *ort*^−^ mutant flies (Schnaitmann et al., 2018) shows that flies lacking Dm9-inhibition (see Figure 2C) still show color opponent processing in R7 that is mediated via the HisCl1-pathway. In contrast, GTACR1-inhibition of Dm9 prohibited color opponent processing in R7 in flies with intact HisCl1 signaling (Figure 4). A significant difference between these two experimental approaches lies in the different activity of Dm9. In the ort^−^ mutant background, Dm9 exhibits aberrant excitatory responses to visual stimuli (Figure 2C). In contrast, optogenetic manipulation induces persistent inhibition. The latter decreases the overall responsiveness of R7 by a so far unknown mechanism (see below) and likely results in a physiological condition in which additional HisCl1-mediated inhibition fails.

By what mechanism do Dm9 cells mediate feedback inhibition to R7/R8 photoreceptors? Optogenetic excitation of Dm9 revealed excitatory input to R7 (Heath et al., 2020). Our experiments with GTACR1 recapitulate photoreceptor-mediated inhibition in Dm9 and reveal a strong decrease in intracellular calcium in all four inner photoreceptor types under these conditions (Figure 5). This suggests that Dm9 do mediate feedback inhibition, most likely through a decrease in the release of glutamate. The latter is suggested by the presence of mRNA for *vGluT* in Dm9 (Davis et al., 2020) and our cell-specific conditional labeling of vGluT protein in Dm9 neurites (Figure 1K). Suitable ionotropic glutamate receptors including EKAR might be expressed in all photoreceptor types of *Drosophila* (Davis et al., 2020). In the lamina, EKAR mediates feedback from lamina intrinsic amacrine cells to R1–R6 photoreceptors which helps to maintain light sensitivity under low ambient light (Hu et al., 2015). Similarly, excitatory signaling from Dm9 to photoreceptors is required to retain sensitivity to low intensity stimuli (Figure 5).

The here used *norpA* and histamine receptor rescue approach is the gold standard that is used in the field to characterize the contribution of specific photoreceptor types (or postsynaptic cells) to visual processing in *Drosophila* (Rister et al., 2007; Gao et al., 2008; Wardill et al., 2012; Wernet et al., 2012; Schnaitmann et al., 2013; Heath et al., 2020). It cannot be excluded that these genetic manipulations elicit certain developmental alterations and compensatory mechanisms. However, in contrast to the vertebrate retina, light detection is seemingly not required for intact development and function of the *Drosophila* visual system. Even rearing flies in complete darkness yields only small behavioral changes (Hirsch et al., 1995; Barth et al., 2010). We propose that reducing light sensitivity to a single type of photoreceptor (*norpA*-rescue) should elicit even smaller changes or no effect at all.

Finally, future studies are required to reveal whether or how the here demonstrated roles of Dm9 in spectral processing and adaptation are relevant for the execution of intact visually guided behavior. We speculate that, in the absence of glutamatergic signaling from Dm9 to inner photoreceptor terminals, spectral and other visual behaviors should in particular be affected at low intensities of ambient light.

### Do *Drosophila* Dm9 cells functionally resemble vertebrate horizontal cells?

Dm9 cells have been postulated as “horizontal-cell-like” neurons due to their anatomical features, positioning, connectivity in the peripheral visual system, findings from calcium imaging, and predictions generated by a computational model (Davis et al., 2020; Heath et al., 2020; Uhlhorn and Wernet, 2020). Our calcium recordings demonstrate that Dm9 cells indeed provide important contributions to spectral inhibition in all four R7/R8 photoreceptor types and corroborate pivotal constraints of the above mentioned model. The global decrease in the calcium responses and shift to higher light sensitivity that we observed in R7/R8 terminals upon optogenetic inhibition of Dm9 further suggest that Dm9 have an additional role in adaptation. Dm9 and horizontal cells have similar positions in the visual circuitry, accomplish multiple tasks in early visual processing and therefore share more functional commonalities than previously expected.

## Data availability statement

The raw data supporting the conclusions of this article will be made available by the authors, without undue reservation.

## Ethics statement

The manuscript presents research on animals that do not require ethical approval for their study.

## Author contributions

CS: Conceptualization, Data curation, Formal analysis, Investigation, Methodology, Software, Supervision, Validation, Visualization, Writing—original draft, Writing—review & editing. MP: Investigation, Methodology, Software, Writing—review & editing. PM: Data curation, Investigation, Visualization, Writing—review & editing. LS: Data curation, Investigation, Visualization, Writing—review & editing. VO: Methodology, Resources, Writing—review & editing. DR: Conceptualization, Funding acquisition, Project administration, Resources, Supervision, Writing—original draft, Writing—review & editing.
