# Supplementary material for: Horizontal-cell like Dm9 neurons in Drosophila modulate photoreceptor output to supply multiple functions in early visual processing
Source: Front Mol Neurosci. 2024 May 15;17:1347540. doi: 10.3389/fnmol.2024.1347540 (PMC11133737; doi:10.3389/fnmol.2024.1347540)
Supplement: Supplementary file 2 [file Data_Sheet_1.docx]

Supplementary Material

Horizontal-cell like Dm9 neurons in *Drosophila* modulate photoreceptor output to supply multiple functions in early visual processing

# Supplementary Figures

**
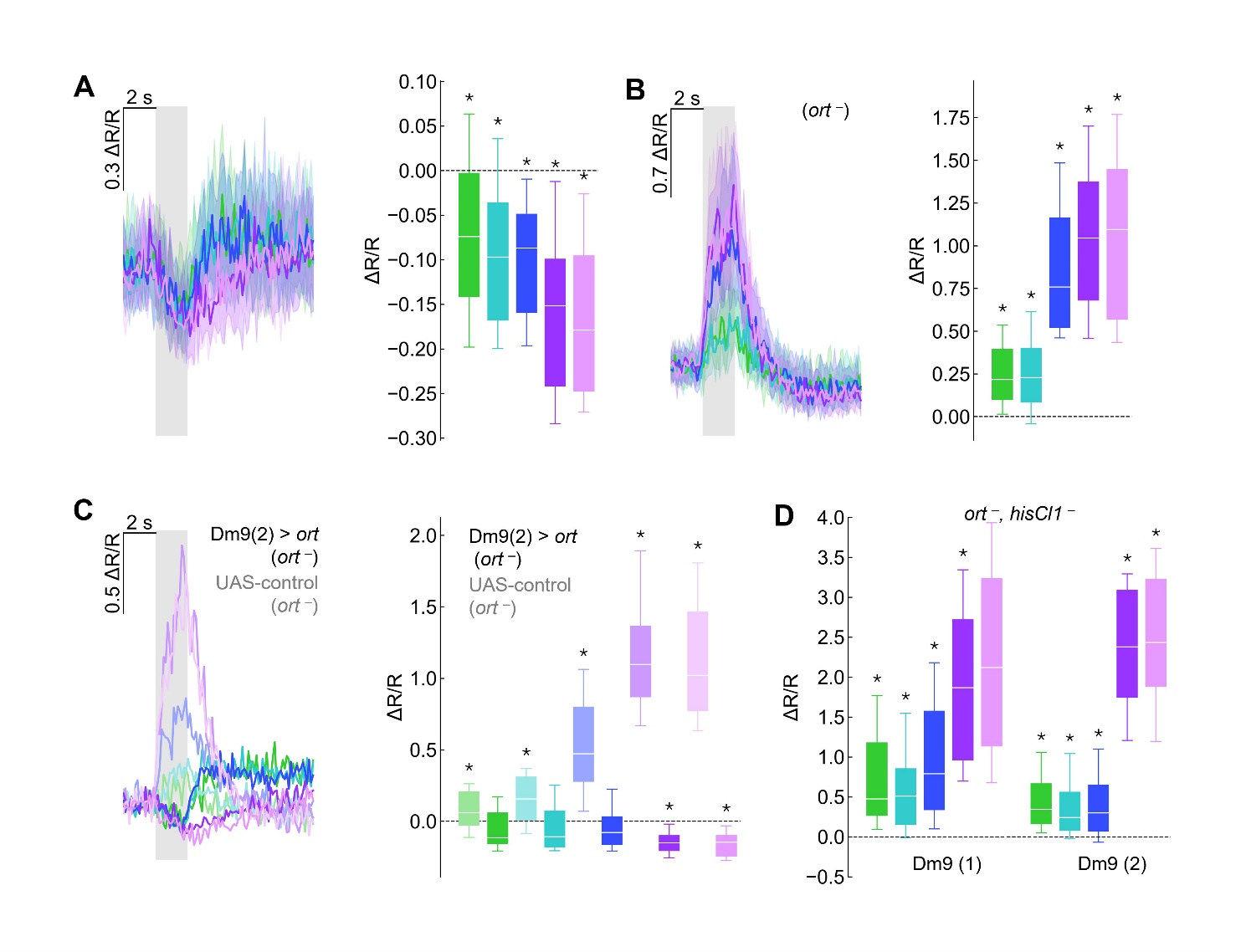
Figure S1. Visually evoked responses in Dm9 in *ort ^–^* mutant and *ort* ^–^, *hisCl1* ^–^ double mutant flies.**

(A) Responses in Dm9 to visual stimuli using another Dm9-GAL4 driver line as in Figure 2, ^*^p < 0.05, Wilcoxon test.

(B) Responses in Dm9 to visual stimuli in *ort^–^* mutant flies using another Dm9-GAL4 driver line as in Figure 2, ^*^p < 0.05, Wilcoxon test.

(C) Responses in Dm9 to visual stimuli in flies with *ort* rescue in Dm9 (Dm9 > *ort*, *ort^–^*) and UAS-control flies (UAS-ort, *ort^–^*, no GAL4) using another Dm9-GAL4 driver line as in Figure 2, ^*^p < 0.05, Wilcoxon test. Responses to the same color stimuli differed between genotypes, p < 0.05, Mann-Whitney U test.

(D) Responses in Dm9 to visual stimuli *ort^–^* ,*hisCl1^–^* double mutant flies using two different Dm9-GAL4 drivers, ^*^p < 0.05, Wilcoxon test.

In (A)–(D), the same type of plots as in Figure 2. All stimuli were shown at maximum intensity (10^3^ a.u.). For genotypes and n recordings, see Table S1.


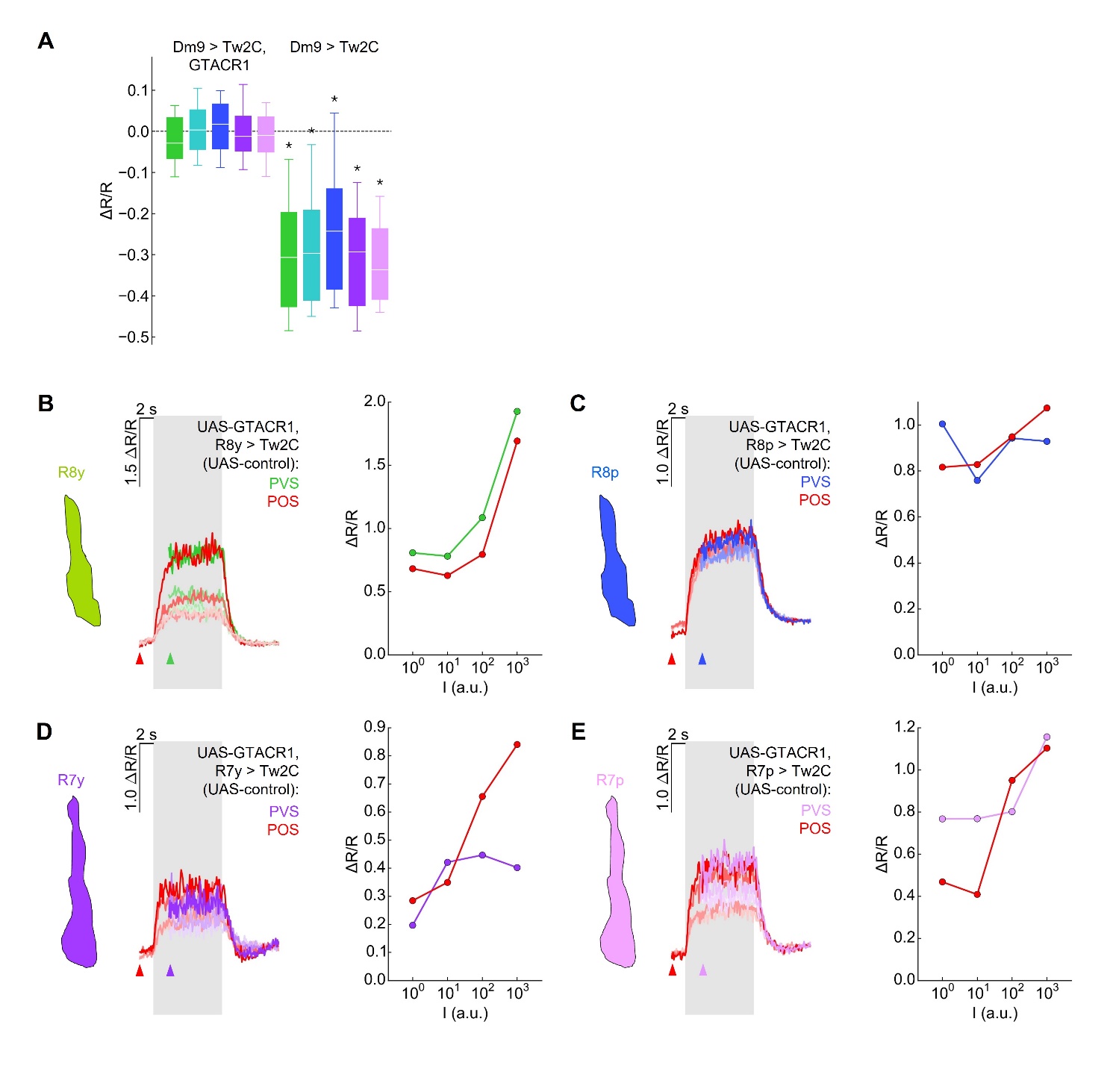
**Figure S2. Optogenetic hyperpolarization of Dm9 decreases the responses in R7/R8 photoreceptor terminals and shifts their sensitivity towards higher stimulus intensities - UAS-controls for experiments in Figures 5A-D.**

(A) Quantified responses from data in Figure 2C, ^*^p < 0.05, Wilcoxon test.

(B) Responses in R8y to different intensities of green stimuli (10^0^ –10^3^ a.u.) in UAS-control flies (harboring UAS-GTACR1 but no GAL4). Same experimental conditions as in Figures 5A-D. In one set of experiments, the onset of calcium imaging and optogenetic stimulation (elicited by the two-photon laser used for calcium imaging) precedes visual stimulus presentation (red; ‚preceding optogenetic stimulation’, POS; onset of laser scanning indicated by red arrow head). In the second set of experiments, the visual stimulation precedes the onset of calcium imaging and optogenetic stimulation (green; ‚preceding visual stimulation‘, PVS; onset of laser scanning indicated by colored (green) arrow head). Gray box indicates visual stimulation period. The magnitude of the responses correlates with stimulus intensity. (left) Time course of responses. (right) R8y average responses to the color stimuli presented at four different intensities (10^0^ –10^3^ a.u.) calculated between 2 s and 3 s after visual stimulus onset (responses with and approx. without Dm9 inhibition in Figures 5A-D). Dm9 Responses to the same intensity stimuli did not differ between experimental groups, p < 0.05, KruskalWallis H test; p > 0.05, post hoc Mann-Whitney U tests).

(C) Same as in (B) for R8p with blue stimuli. Responses to the same intensity stimuli did not differ between experimental groups, p > 0.05, KruskalWallis H test).

(D) Same as in (B) for R7y with UV_l_ stimuli. Responses to the same intensity stimuli did not differ between experimental groups, p < 0.05, KruskalWallis H test; p > 0.05, post hoc Mann-Whitney U tests).

(E) Same as in (B) for Ryp with UV_s_ stimuli. Responses to the same intensity stimuli did not differ between experimental groups, p < 0.05, KruskalWallis H test; p > 0.05, post hoc Mann-Whitney U tests).

For genotypes and n recordings, see Table S1.
